# The Need for Laparoscopic Ovarian Transposition in Young Patients with Cervical Cancer Undergoing Radiotherapy

**DOI:** 10.1155/2013/173568

**Published:** 2013-12-03

**Authors:** Hariyono Winarto, Eva Febia, Gatot Purwoto, Laila Nuranna

**Affiliations:** Gynecologic Oncology Division, Department of OBGYN, Faculty of Medicine, University of Indonesia, Gedung A lt. 3, Medical Staff Wing Dr. Cipto Mangunkusumo Hospital, Jakarta 10430, Indonesia

## Abstract

Maintaining the quality of life by preserving ovarian function in premenopausal patients with cervical cancer undergoing radiation is crucial. This can be accomplished with a simple and safe laparoscopic ovarian transposition procedure. This procedure aims to move the ovary out of the irradiation field, protecting it from direct radiation and irreversible damage and preserving its function. However, this procedure is often forgotten and seldom offered to patients. This review aims to lay stress on and reconsider the importance of laparoscopic ovarian transposition as a simple, safe, and extremely useful procedure. The biological effects of radiation are described briefly and several studies are evaluated, which reveal that this procedure has more benefits than risks.

## 1. Introduction

Cervical cancer is one of the commonest malignancies in Indonesian women. The annual incidence is 9.25 per 100000 population. Due to advances in screening and treatment, patients with cervical cancer are being diagnosed at a younger age and earlier stage of the disease. Approximately half the patients are premenopausal and under 45 years old. Radiotherapy, which constitutes almost 80% of all cancer treatment modalities, causes irreversible ovarian damage and leads to premature menopause, which affects the quality of life. Young women with cervical cancer who are irradiated often have to suffer the long-term consequences of ovarian failure. In addition to curing those patients, maintaining their quality of life is important for a gynecologist. Improved quality of life is significantly associated with improved survival in patients with cervical cancer [1–8].

A simple procedure for preventing radiotherapy-induced ovarian damage is laparoscopic ovarian transposition. This procedure has not been implemented widely even though many studies have revealed its benefits and efficacy. This review assesses the use of ovarian transposition as an effective method for preserving ovarian function in young patients with cervical cancer undergoing radiotherapy and compares the benefits of ovarian transposition performed via laparoscopy and via laparotomy [9–13].

## 2. The Biological Effect of Radiotherapy and the Fate of Retained versus Transposed Ovaries

**Figure 1:**
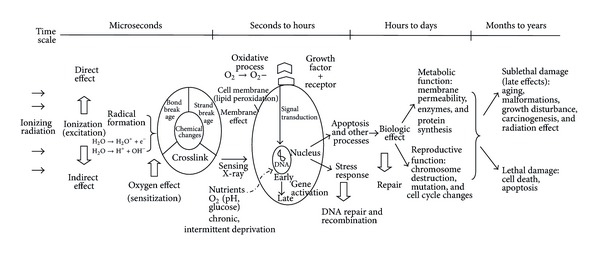
Fate of irradiated cell. Schematic representation of the various processes that take place after cell irradiation [14–16].

Radiotherapy aims to deliver a precisely measured dose of radiation to a defined tumor volume with minimum possible damage to the surrounding normal tissue. Ionizing radiotherapy interacts with DNA. The initial DNA damage leads to a cascade of biologic events that cause lethality when the cells attempt to divide (mitotic death) or programmed cell death (apoptosis), as well as sublethal damage that leads to aging, malformations, and malfunction (Figure 1) [14, 15].

The human ovary contains a finite number of ovarian follicles, which are vulnerable to DNA damage from radiotherapy. The degree and persistence of ovarian damage and the suppression of its function are related to the patient's age and the dose of radiation delivered to the ovaries. After the ovaries are exposed to ionizing radiation, if the dose of radiation exceeds the lethal dose, most of the primordial follicles and granulose cells will die in microseconds. Some of those follicles and cells experience sublethal damage, leading to accelerated functional failure. Only a small number would escape the damage, undergo repair, and still have their function. Pyknotic granulose cells would be seen soon after irradiation, and with sufficient destruction of the granulose cells, the follicle would become atrophic. There would be a loss of the cortical stromal cells, and in time, the cortical volume would be replaced by collagen [6, 12, 13, 15, 17, 18].

The cutoff dose for radiation-induced ovarian failure is around 8–20 Gy. A dose >8 Gy causes permanent ovarian damage in almost all patients older than 40 years. A dose >20 Gy causes permanent sterility in patients of any age, with complete or near complete disappearance of the primordial follicles. Radiotherapy used in cervical cancer treatment consists of high-dose external radiotherapy and brachytherapy. The dose of radiotherapy for cervical cancer should be lethal to the cervical tumor tissue. It ranges from 45 Gy to as high as 90 Gy, exceeding the lethal dose for ovarian follicles, and results in permanent damage and loss of ovarian functions in all patients of any age, unless some interventions are applied. Transposing the ovaries is a method of minimizing ovarian follicle exposure to radiation [12, 13, 19].

## 3. Rationale, Benefits, Complications, and Indications of Ovarian Transposition

**Figure 2:**
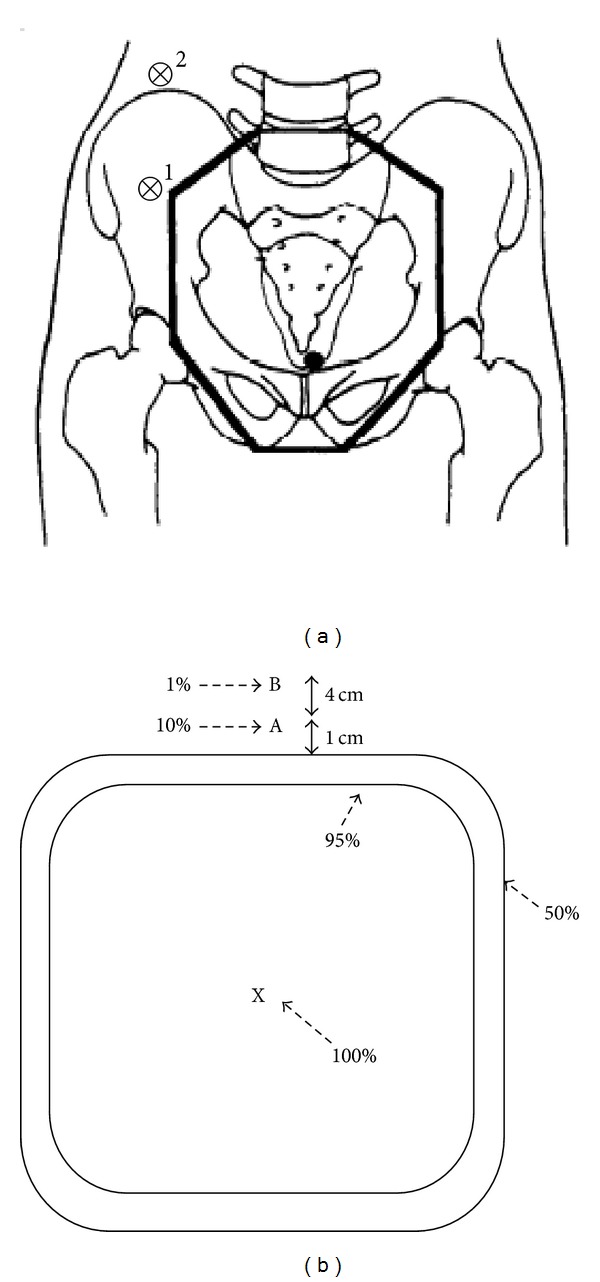
(a) Diagram showing the (AP-PA) whole pelvic radiation therapy field relative to the position of the transposed ovary. Position 1 represents the suboptimal placement of the ovary; position 2 represents the optimal placement of the ovary in terms of maintaining ovarian function. (b) Diagram of a dose distribution. The dose at point A is 10% of the dose at the center of the field (point X, 100%). The dose at point B is 1% of the dose at point X. Therefore, if the prescribed dose was 45 Gy, the dose is 4.5 Gy at point A and 0.45 Gy at point B [13, 25].

Transposing the ovary away from the radiation field can be done surgically at the time of radical hysterectomy or before radiotherapy. In patients with cervical cancer, the ovaries are transposed or moved to the lateral side of the abdomen above the pelvic inlet to place them far enough from external pelvic radiation exposure or brachytherapy. If the ovaries are transposed laterally, about 3 cm above the pelvic inlet, they will receive only 1%–10% of the total radiotherapy dose. If the total radiotherapy dose is 45 Gy, then the dose received by the transposed ovaries is only 0.45–4.5 Gy, whereas the retained ovaries could receive 50%–70% of the total dose, which is 20–32 Gy [13, 20–24] (Figure 2).

Successfully preserving ovarian function depends on the distance between the transposed ovaries and the edge of the radiation field. Therefore, the ovaries should be transposed as laterally and as cranially as possible from the pelvic brim. However, attention should be paid to avoid torsion and extension of the ovarian vessels, which may reduce blood supply to the ovaries [26, 27].

Lateral ovarian transposition can be performed towards the subcutaneous tissue of the flank or the paracolic gutter. However, attaching the ovary to the flank produces more pain complaints than lateral transposition of the ovaries into the paracolic gutter, which is more widely accepted and results in minimal complications (Table 1). Lateral ovarian transposition to the paracolic gutter lateral to the ascending or descending colon is considered a simple standard procedure and can be done laparoscopically [23, 25, 28–32].

Studies on lateral ovarian transposition show that it is 44%–85% effective in preserving ovarian function and that complications such as symptomatic ovarian cyst formation range from 0% to 27%. Symptomatic cyst formation occurs more frequently after lateral ovarian transposition to the subcutaneous adipose tissue (20%–27%) than after lateral ovarian transposition to the paracolic gutter. Ovarian metastasis is rare (0%–1.2%) but is reported. A case of abdominal trocar insertion metastasis after laparoscopic lateral ovarian transposition in a patient with cervical cancer, adenocarcinoma stage IIB was reported. The incidence of trocar insertion metastasis is <1%. As lateral ovarian transposition to the paracolic gutter is a simple and safe procedure for preserving ovarian function, its benefits outweighs the risks of complications [23–25, 28–35] (Table 1).

Lateral ovarian transposition is indicated in young patients with cervical cancer after radical hysterectomy, after neoadjuvant chemotherapy, before radiotherapy, or before concurrent chemoradiotherapy. Some studies showed that the addition of cisplatin in a low dose of 50 mg/m^2^, used in neoadjuvant chemotherapy or concurrent chemoradiotherapy, did not significantly alter ovarian function. However, adding cisplatin concurrently as radiotherapy functioned as a radiosensitizer and was proven to have better results in some studies. The addition of vincristine and bleomycin in low doses of 1 mg/m^2^ and 25 mg/m^2^, respectively, as neoadjuvant chemotherapy did not alter ovarian function either. Lateral ovarian transposition is still used in young patients with cervical cancer after they receive chemotherapeutic agents such as cisplatin, vincristine, and bleomycin in low doses as used in neoadjuvant chemotherapy or concurrent chemoradiotherapy [24, 25, 33, 36–42].

## 4. Uterine Transplantation and Surrogacy

Besides maintaining ovarian function, ovarian transposition can be used in women who wish to maintain their fertility and reproductive function. For patients with cervical cancer who have undergone radiotherapy that extended to the uterus, and/or hysterectomy, uterine transplantation and surrogacy after ovarian transposition are alternatives. However, uterine transplantation has succeeded only in the animal research setting, and only one failed attempt of human uterus transplantation has been reported. Moreover, uterus transplantation and surrogacy are still under ethical debate concerning both the recipient and the donor's reproductive rights [43–46].

## 5. Laparoscopy versus Laparotomy Unilateral Ovarian Transposition

Lateral ovarian transposition is a simple procedure that can be performed laparoscopically. Laparoscopic ovarian transposition is superior to laparotomy. Many studies showed that laparoscopic ovarian transposition, like other minimally invasive procedures, produced less risk of adhesion, inflammation, and shortened length of hospital stay in addition to reduced recovery time, resulting in fewer delays in radiotherapy compared with laparotomy. Since laparotomy necessitates a longer postoperative recovery time, it significantly delays radiotherapy. In some cases, the transposed ovary has returned to the previous position due to the delay, resulting in failure to preserve the ovaries [9, 10, 33].

Laparoscopic ovarian transposition can be performed in a day and the patient is sent for radiotherapy. The minimal postoperative wound will not affect mobility and functional activity, as well as quality of life in cervical cancer patients. Studies showed that the risk of adhesion and cyst formation was less with laparoscopic ovarian transposition compared with laparotomy [9, 10, 28, 29, 33, 34].

Laparoscopic ovarian transposition can be performed on either ovary. Studies show that steroid hormone production from only one ovary is enough to prevent ovarian function failure. Clough et al. [30] and Giacalone et al. [47] showed that unilateral right ovarian transposition effectively preserves ovarian function in 85% of subjects. Additionally, unilateral ovarian transposition of the right ovary to the paracolic gutter, as high as the subhepatic region, is technically easier, resulting in fewer complications.

It is important to measure ovarian reserve in patients with cervical cancer aged 41–49 years and in those who have received neoadjuvant chemotherapy before laparoscopic ovarian transposition. To test the ovarian reserve, the menstruation cycle should be assessed and levels of the follicle stimulating hormone (FSH), estradiol, and anti-Müllerian hormone (AMH) should be measured. The AMH has an added value since its level is independent of the menstrual cycle, so its assessment can be used in patients who have undergone radical hysterectomy or experienced acute amenorrhea due to neoadjuvant chemotherapy. In some studies, the cutoff value of the AMH indicating good ovarian reserve varied, such as ≥0.3 ng/mL, ≥0.5 ng/mL, and ≥1.4 ng/mL [48–50].

## 6. Conclusion

Laparoscopic ovarian transposition is a simple, safe, effective, but often forgotten, procedure for young premenopausal patients with cervical cancer who are undergoing radiotherapy. We believe this procedure should be offered to all young premenopausal patients with cervical cancer undergoing radiotherapy to preserve their ovarian function. However, further studies to evaluate the efficacy of laparoscopic ovarian transposition in our center are still needed.

## Figures and Tables

**Table 1:** Studies reporting the outcome of lateral ovarian transposition in patients with cervical cancer.

Author	Procedure	Number of subjects	Position of transposed ovary/ovaries	Therapy	Outcome
Feeney et al. [29]	Lateral ovarian transposition following hysterectomy	28	To the paracolic gutter	RT/RH + RT	Ovarian preservation was achieved in 14/28 (50%) patients

Fujiwara et al. [28]	Subcutaneous ovarian transposition following hysterectomy	27	To the fascia of the abdominal tissue	RT + RH	Only 12 patients (44%) had normal ovarian function

Anderson et al. [23]	Ovarian transposition	82	Sutured to the posterior peritoneum, above the pelvic brim at the level of the lower pole of the kidney	RT	Ovarian preservation was achieved in 53% of subjects. Painful ovarian cyst occurred in 20% of cases. There was one case of ovarian metastasis (1.2%)

Huang et al. [24]	Laparoscopic bilateral ovarian transposition	14 (<45 years old)	To a high anterolateral position, 3-4 cm above the umbilical line	CCRT/RT/RH + RT/NCT + RH + RT	No intraoperative or postoperative complications occurred. No metastasis was observed. All patients tolerated the procedure. Seven of the 14 patients (50%) developed ovarian failure, shown by the elevation of FSH level

Morice et al. [31]	Bilateral ovarian transposition	107 (21–42 years old)	To the paracolic gutter (laparotomy, 102 cases; laparoscopy, 5 cases)	RT/RH + RT	One case (1%) with ovarian metastasis. No other postoperative complications occurred. Ovarian function preservation was achieved in 83% of patients

Morice et al. [33]	Bilateral ovarian transposition	24	To the paracolic gutter (laparoscopy)	RT/RH + RT/NCT + RT + RH	Ovarian preservation was achieved in 79% patients; three pregnancies were obtained

Chambers et al. [25]	Lateral ovarian transposition (by laparotomy)	34	Below and above the iliac crest	RT/RH + RT/CCRT	Ovarian preservation was achieved in 71%. Symptomatic ovarian cyst occurred in 18% of cases

Clough et al. [30]	Laparoscopic unilateral (right) ovarian transposition	20	To the paracolic gutter	RT	There were (18/20; 85.3%) cases with normal ovarian function. No postoperative complication was observed

van Eijkeren et al. [34]	Lateral ovarian transposition following hysterectomy	18	To the abdominal sidewall at the level of the lowest rib	RT	Ovarian preservation was achieved in 13/18 (72%) patients

CCRT: concurrent chemotherapy radiotherapy (adding cisplatin as radio sensitizer with a dose of 50 mg/m^2^ weekly for 6 courses); RT: radiotherapy; RH: radical hysterectomy; NCT: neoadjuvant chemotherapy (combined cisplatin 50 mg/m^2^, vincristine 1 mg/m^2^, and bleomycin 25 mg/m^2^ in an interval of 10 days, 3 courses in total).
